# Evaluation of a train-the-coach program in the context of a complex intervention for diabetes type 2 and coronary heart disease patients (P-SUP study)

**DOI:** 10.3389/fmed.2025.1577341

**Published:** 2025-07-11

**Authors:** Isabel Hamm, Aliza Lehmann, Angeli Gawlik, Yeliz Nacak, Julia Timmke, Christian Funke, Stefan Wilm, Lisa Giesen, Marcus Redaèlli, Chloé Chermette, Frank Vitinius

**Affiliations:** ^1^Department of Psychosomatic and Psychotherapy, Faculty of Medicine and University Hospital of Cologne, University of Cologne, Cologne, Germany; ^2^Department of Health and Social Psychology, Institute of Psychology, German Sport University Cologne, Germany; ^3^Centre for Health and Society (CHS), Institute of General Practice (IFAM), Medical Faculty of the Heinrich-Heine-University Dusseldorf, Dusseldorf, Germany; ^4^Faculty of Medicine and University Hospital of Cologne, Institute for Health Economics and Clinical Epidemiology, University of Cologne, Cologne, Germany; ^5^Department of Psychosomatic Medicine, Robert-Bosch Hospital, Stuttgart, Germany

**Keywords:** train the trainer, telehealth, coaching, diabetes, coronary heart disease

## Abstract

**Introduction:**

Health coaching can address various health-related issues to improve health parameters and is used to manage chronic diseases, such as type 2 diabetes mellitus (T2DM) or coronary heart disease (CHD). For high-quality coaching, training as preparation is important. However, few training programs have been evaluated so far. In this study coaches should provide telehealth coaching to patients with T2DM and/or CHD within a peer support program to increase overall health status. In this paper the training for telehealth coaches was assessed with qualitative and quantitative methods.

**Materials and methods:**

The training of the coaches (*n* = 4) consisted of 38 h with 11 units on health action process approach (HAPA), motivational interviewing (MI), nutrition and physical activity, the self-determination theory (SDT) and self-efficacy as well as practice coaching. The training was evaluated based on the Kirkpatrick model, quantitatively with an evaluation survey and a knowledge test on a 7-point Likert scale and qualitatively semi-structured interviews, with emphasis on the qualitative evaluation. A follow-up assessment 2.5 years after the training was conducted.

**Results:**

In the evaluation survey the overall training was rated as highly informative (*M* = 6.75) instructive (*M* = 6.5), practical (*M* = 6.25) and satisfactory (*M* = 6.25). The knowledge test was completed with 76 to 93% correct answers. The qualitative interviews revealed the usefulness of the MI and HAPA units. The practice coaching with actor patients as well as among each other were highlighted as important. Collectively, in-person training was favored by the participants compared to online training. The follow-up assessment emphasizes the results.

**Discussion:**

Overall, the evaluation showed the training was well accepted and perceived as useful in preparation for the coaching activity.

## 1 Introduction

Health coaching is a patient-centered intervention that helps patients achieve health goals through education, guidance and motivation. It employs methods like empathic listening, patient-chosen goals, and evidence-based practices to foster intrinsic motivation, self-efficacy, and resilience (1, 2). Health coaching addresses various health-related issues, including chronic disease management, lifestyle changes, stress management and behavior modification (3).

For patients with chronic illness, in the context of health interventions, coaching refers to a structured, collaborative process in which trained professionals support individuals in setting and achieving health-related goals by enhancing motivation, self-awareness, and behavior change skills. This is distinct from counseling or training, which may focus more narrowly on emotional support or skills instruction, respectively. This approach aims to improve patients’ quality of life and positively impact disease progress and symptoms (4). High-quality coaching requires thorough training to equip coaches with necessary knowledge and skills (5). Practical experience and exchange with other coaches enhance learning success (6). The role of the coach is crucial for the coaching success (7–9). However, since “coach” is not a protected title, thorough training and evaluation are essential to ensure competent, method-based coaching. Few training programs have been evaluated so far (6, 10–12). Programs such as “PEACH” [Patient Engagement and Coaching for Health; (13)], “diabetescoach” (14), “Healthy at Home” (15) and other structured interventions for T2DM and CHD patients have demonstrated improvements in glycemic control, medication adherence, and patient empowerment. These programs typically involve multiple coaching sessions over a span of several weeks or months, and rely on standardized protocols delivered by trained coaches. Training programs for health coaches differ considerably in terms of curriculum, duration, and delivery methods. Some focus on communication techniques, motivational interviewing, and health behavior theories, while others provide specialized content for chronic conditions such as diabetes or cardiovascular disease. Implementation often includes a combination of classroom instruction, practical exercises, and supervised coaching sessions. However, empirical evidence regarding the long-term effectiveness of these training programs on patient outcomes remains limited.

The telehealth coaching intervention is part of a personalized self-management support program (P-SUP) in Germany (16). This intervention is integrated into a broader, multi-component disease management framework (P-SUP). The coaching intervention is grounded in a unique combination of theoretical models and emphasizes practice-based learning through actor-patient simulations and peer coaching. Unlike previously evaluated programs, this intervention includes a long-term follow-up and evaluates the transfer of learned competencies into actual coaching practice. This study aims to evaluate the training of coaches for their telehealth coaching activities, using qualitative and quantitative methods to develop a comprehensive train-the-coach-concept in the health sector.

Specifically, the study aims to:

(1) Assess the subjective satisfaction and perceived utility of the training program.
(2) Evaluate the acquisition of relevant coaching knowledge and skills.
(3) Investigate how well the acquired competencies are applied in coaching practice.

## 2 Materials and methods

### 2.1 Concept of the evaluated training

In preparation for the telehealth coaching of the personalized self-management support program (P-SUP) (16) a coaching training took place, which is evaluated in this paper.

P-SUP is a comprehensive intervention for patients in a German disease management program targeting the health improvement of type 2 diabetes mellitus (T2DM) and/or coronary heart disease (CHD) patients. P-SUP offers peer support group meetings for 18 months, personalized feedback, a web portal with support modules, and telehealth coaching for patients with low health literacy and/or low activation level. The telehealth coaching includes 13 telephone sessions, scheduled for 20 min, with the initial and final sessions lasting 30 min with intervals between sessions Coaches received weekly supervision during the coaching process.

In preparation for telehealth coaching, all coaches received extensive training. The development of the training, explained in a concept paper (17), was built on different theories and approaches, including the health action process approach (HAPA) (18), motivational interviewing (MI) (19), the self-determination theory (SDT) (20) and self-efficacy (20). Units on physical activity, healthy nutrition, and practice coaching with actor patients and among coaches were included. Additionally, coaches participated in a communication training for physicians (21). This communication training was not part of the coaching training and the participation of it is not evaluated.

Table 1 illustrates an overview of the content of the training program. HAPA explains the processes in initiating and maintaining behavior change (18), while SDT describes the need for supportive environments for lasting behavior change (20). MI increases motivation and readiness to change behavior (19), with self-efficacy being central to both HAPA and MI (22). Sixteen tools were developed for coaching sessions based on these theories (17). The 38-h training consisted of 11 units, each lasting 2–7h, combining presentations and interactive exercises (e.g., using cards to recreate the HAPA model as a group work or categorizing case studies in the HAPA model). Practice coaching included guided sessions with actor patients and peer sessions without trainers. Both forms are effective for learning and refining communication skills (23).

**TABLE 1:** Overview of the training contents.

Training unit	Topic	Duration (hours)
Unit 1	Health action process approach (Theory and practice)	7
Unit 2	Motivational interviewing (principles, attitude and techniques)	4
Unit 3	Application and practice of MI techniques with HAPA tools	4
Unit 4	Application and practice of HAPA tools and MI techniques with actor patients	3
Unit 5	Structure of TC, presentation of future work materials for the documentation of coaching sessions	7
Unit 6	Nutrition knowledge	3
Unit 7	Physical activity knowledge	3
Unit 8 + 9	Self-determination theory and self-efficacy	2
Unit 10 + 11	Renewed practice coaching with actor patients	2.5 each

### 2.2 Evaluation of the training—study design

The training was evaluated using an evaluation survey, a knowledge test and qualitative interview. The focus was on the qualitative interviews. The evaluation survey and knowledge test were anonymously collected, the qualitative interview was anonymized through transcription.

The evaluation was based on the Kirkpatrick model which is empirically proven to evaluate trainings (24), which includes four levels. Level 1 *Reaction* (evaluated with evaluation survey and interviews) measures participants’ perceptions of the training’s usefulness, engagement, and relevance. Level 2 *Learning* (evaluated with knowledge test and interviews) assesses the acquisition of knowledge, skills, attitudes, confidence, and commitment. Level 3 *Behavior* (determined through interviews, as initial sessions had already begun) evaluates the application of learned skills in the workplace. Level 4 *Outcomes* examines the training’s impact on intended outcomes (24). Level 4 *Outcomes* could not be evaluated due to the complexity of the multi-component design of the P-SUP study.

The evaluation of the training was complemented by a follow-up assessment after the coaching intervention. An interview, the knowledge test and the evaluation form were used again. The interview was adapted for the follow-up assessment, while the knowledge test and evaluation form remained unchanged. Due to the small sample size, this study is exploratory. The focus is on qualitative insights rather than quantitative measurements.

### 2.3 Measurements

#### 2.3.1 Evaluation survey

The evaluation survey aimed to examine the acceptance and perceived helpfulness of the training (first level of Kirkpatrick’s evaluation). Coaches assessed how informative, instructive, practical, and satisfying several components of the training were:

- Informative: Amount of knowledge conveyed.
- Instructive: Quality of guidance and direction provided.
- Practical: Applicability and usefulness of training content in real coaching situations.
- Satisfying: Level of fulfillment experienced.

The components assessed included individual units and techniques (theories, actor patients, videos, feedback from participants/trainers), the didactic approach, the training atmosphere (commitment of participants, working atmosphere of trainers, competencies of trainers) and overall impression. The survey used a 7-point Likert scale for 14 questions from 1 (no agreement at all) to 7 (fully agreement), and was developed by the author team based on a previous communication training evaluation (21). The survey was conducted 6 months post-training.

#### 2.3.2 Knowledge test

The knowledge test aimed to determine the retention of acquired knowledge and skills during the training. It covered the following topics and their application: HAPA, MI, self-efficacy and SDT. The test included multiple-, forced-choice (11 questions) (*Which of the questions are not suitable according to the principle of motivational interviewing?*) and open-ended questions (10 questions) (*Describe self-efficacy in your own words.*) asking about the theoretical and practical background of the coaching concept. Coaches worked on two coaching cases and identified HAPA tools. Each correctly answered task was given one point (if three out of four asked aspects were correct, 0.75 points were given). The knowledge test was developed by the authors. The test was conducted alongside the evaluation survey, 6 months post-training.

#### 2.3.3 Qualitative interview

The qualitative interview captured the coaches’ experiences with the training and evaluated the utility and application of the knowledge gained. Semi-structured guidelines allowed for flexibility and standardization. The structured content analysis was applied (25). The guiding questions followed the three levels of the Kirkpatrick model (Reaction, Learning, Behavior):

- Reaction: Coaches’ overall feelings about the training, perception of individual content, and practice coaching.
- Learning: What coaches internalized, found useful, and what was missing.
- Behavior: Confidence and competence in conducting sessions and observed challenges.

The order of questioning was flexible and allowed free responses. Each interview lasted approximately 1.5 h and was conducted by three authors (CF, LG, MR), who were not supervisors and did not conduct the training to ensure free expression. Coaches consented to the conduction and audio recording, which occurred 1-year post-training, after at least one coaching session took place. One interview was repeated 6 months later, due to technical difficulties, from memory, the interviews did not differ fundamentally.

### 2.4 Statistical analyses

The evaluation survey and knowledge test were analyzed descriptively using SPSS [Version 29.0.2.0 (26)].

The interviews were coded using MAXQDA (27) and transcribed per Dresing and Pehl’ rules (1–5, 8–14) (28). Deductive categories from the Kirkpatrick model and guiding questions were established, discussed, and approved by all authors. Two independent coders began coding one qualitative interview, discussing codes after short segments to ensure consistency Overall, 671 codes were assigned across 20 categories and 40 subtopics (see coding guide: Supplementary Appendix A).

The authors condensed the code system, achieving a good intercoder reliability [Cohen’s kappa (29) of 0.70]. The codes were then summarized after the qualitative content analysis.

### 2.5 Participants

Participants were selected based on the following inclusion criteria: (1) availability for full participation in the training and follow-up activities, (2) relevant educational or professional background in health, nutrition, or exercise science, and (3) willingness to engage in telehealth coaching activities. Exclusion criteria included lack of German language proficiency.

Four coaches were hired for telehealth coaching because four were sufficient to oversee the patients, so the sponsor was asked to provide a corresponding number of jobs in this project. They underwent the training to prepare for the coaching. The prior knowledge of the coaches ranged from a sole health science background to previous coaching experience. In Table 2 the characteristics of the coaches are shown. Three coaches took part in the follow-up assessment (see Table 2 for more information).

**TABLE 2:** Characteristics of the coaches.

Coach	Sex	Age	Educational qualifications
Coach 1*	Female	34	Sports science, B.Sc. Nutritional science, B.A.
Coach 2	Female	38	Sports science, Diploma
Coach 3*	Female	59	State certified dietician Prevention and health psychology, B.A.
Coach 4*	Male	50	Sports science, Diploma

The coaches marked with * took part in the follow-up assessment.

## 3 Results

### 3.1 Results of the evaluation survey

**FIGURE 1:**
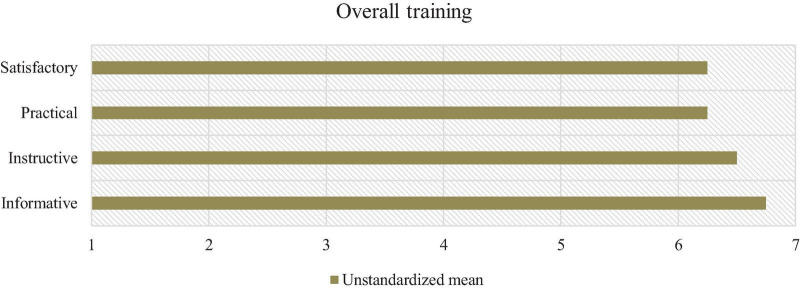
*N* = 4. Means of the overall training evaluation.

The evaluation survey captured the subjective perceptions of the coaches about the informative, instructive, practical, and satisfactory conditions of various components of the training. The overall training was averagely rated as highly informative (mean = 6.75; SD = 0.5), instructive (mean = 6.5; SD = 1), practical (mean = 6.25; SD = 0.5), and satisfactory (mean = 6.25; SD = 0.95; Figure 1). The HAPA training session was rated as most informative, instructive, practical, and satisfactory (Table 3).

**TABLE 3:** Descriptive statistics of the overall training and training units of the evaluation.

		Min	Max	Mean	SD
Overall	Informative	6	7	6.75	0.5
	Instructive	5	7	6.5	1
	Practical	6	7	6.25	0.5
	Satisfactory	5	7	6.25	0.95
HAPA	Informative	6	7	6.75	0.5
	Instructive	6	7	6.75	0.5
	Practical	6	7	6.5	0.57
	Satisfactory	6	7	6.5	0.57
MI	Informative	6	7	6.25	0.5
	Instructive	5	7	6.5	1
	Practical	7	7	7	0.0
	Satisfactory	6	7	6.75	0.5
Self-efficacy	Informative	6	7	6.25	0.5
	Instructive	5	7	6	0.81
	Practical	5	7	6	0.81
	Satisfactory	5	7	6	0.81
SDT	Informative	6	7	6.33	0.57
	Instructive	6	7	6.33	0.57
	Practical	6	7	6.33	0.57
	Satisfactory	6	7	6.33	0.57
Nutrition	Informative	6	7	6.5	0.57
	Instructive	5	7	6.25	0.95
	Practical	6	7	6.5	0.57
	Satisfactory	5	7	6.25	0.95
Physical activity	Informative	2	5	3.75	1.5
	Instructive	2	5	3.5	1.73
	Practical	2	5	4.25	1.5
	Satisfactory	2	5	3.75	1.5

*N* = 4. HAPA, health action process approach; MI, motivational interviewing; SDT, self-determination theory.

### 3.2 Results of the knowledge test

**FIGURE 2:**
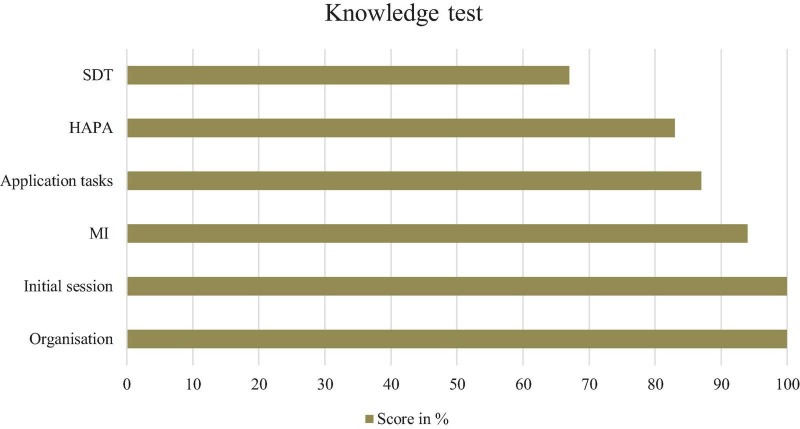
Correctness of the knowledge test. *N* = 4. HAPA, health action process approach; MI, motivational interviewing; SDT, self-determination theory.

The knowledge test assessed the internalized knowledge of the coaches after the training regarding the individual training units (Table 4). The overall knowledge score of the coaches varied between 76 and 93% (Figure 2).

**TABLE 4:** Descriptive statistics of the knowledge test.

	Items	Min	Max	Mean	SD
HAPA	4	2	4	3.33	0.90
MI	4	3	4	3.75	0.5
Self-efficacy	4	2	4	3.06	0.83
SDT	4	2	3.5	2.80	0.79
Organization	4	4	4	4	0.0
Application task	2	1	2	1.75	0.51
Initial session	1	1	1	1	0.0
Overall score (%)	4	76	93	85	7

*N* = 4. HAPA, health action process approach; MI, motivational interviewing; SDT, self-determination theory.

### 3.3 Results of the qualitative interview

Seven themes emerged from the qualitative interviews. The first five address the perception of the training (Level 1), while the last two identify the knowledge and skills acquired (Level 2) and the application in the coaches’ everyday work (Level 3).

#### 3.3.1 Level 1: perception of the training in general

Overall, the training was perceived as informative and insightful and was considered beneficial. Coaches emphasized the interactive design, which balanced imparting knowledge and practical relevance, including practice coaching.

Positive responses by coaches highlighted individual feedback and support from training leaders, clear communication and comprehensive information on the training structure.

Both in-person and online formats during the COVID-19 pandemic were seen as informative, with in-person training being preferred: “Of course, it would always be nicer to do such trainings not online but in person, but the pandemic simply put a spanner in the works” (Interview 3).

Online training was seen as beneficial but more strenuous. Coaches valued the first session in person, as it allowed them to get to know each other better, though some felt uncomfortable with practice coaching in front of peers.

Practice coaching with actor patients was considered an important learning process, increasing coaches’ security and competence. The combination of practice coaching and feedback was particularly helpful. Both male and female actor patients participated which was noted positively. Nonetheless, coaches felt the feedback time was too short and unevenly distributed, leading to some providing feedback via e-mail afterward instead of direct exchanges.

Some coaches mentioned the large time gap between training completion and the beginning of their work as coaches, due to COVID-19 project delays. During this period, coaches practiced coaching among each other weekly for 6 months in online meetings, which they suggested should be a permanent part of the training. This format allowed for longer and more varied practice compared to sessions with actor patients and was perceived as more intense. However, feedback was not as central in these sessions. Coaches wanted more information about other components of the P-SUP intervention.

#### 3.3.2 Level 1: perception of health action process approach

In the first unit, HAPA and the coaching session structure was taught, perceived as fundamental and helpful [see concept paper (17)]. Coaches appreciated the step-by-step explanation of the tools and effectiveness of the tools for time-limited sessions. The interactive delivery, including visual illustrations, case studies, and partner work, enhanced learning and active exchange. However, one coach felt overwhelmed by the amount of content, suggesting case studies to be done at home and presented later to reduce in-session input: “I found it all very exciting, but then also a lot at the end. And that made me feel a bit exhausted” (Interview 2).

#### 3.3.3 Level 1: perception of motivational interviewing

The MI unit was described as interactive and diversified. Coaches liked the interactive elaboration of the communication model, as assembled like a puzzle, which helped them understand and internalize the technique. Online partner work was appreciated, and coaches felt they learned important conversational techniques for coaching sessions. Some even reflected on their general communication behavior. While it made no difference whether the content was taught in-person or online, participating in online training felt unusual. One coach wished there was more time for questions during this unit.

#### 3.3.4 Level 1: perception of nutrition and physical activity

Coaches positively mentioned the teaching of the “plate concept” (30) and WHO (World Health Organization) guidelines. Overall, the content was perceived as being practice-oriented. However, one coach felt it lacked relevance to their upcoming coaching work, providing no new insights and leading to frustration. They suggested sports scientists teach physical activity knowledge to nutritionists and vice versa. Coaches also wanted more information about the P-SUP intervention to understand the full context of what coachees were receiving alongside telehealth coaching.

#### 3.3.5 Level 1: perception of self-efficacy and SDT

The self-efficacy and SDT unit was well remembered and motivating for coaches to explore further. Coaches found it insightful to understand their impact on the coaching process and coachees’ self-efficacy. Two coaches emphasized the theoretical background of self-efficacy as important for coaching sessions. However, another coach felt this unit was less relevant to telehealth coaching. Another saw digital knowledge transfer as an opportunity to promote their own self-efficacy, while one criticized the proportion of frontal teaching, especially in the online format.

#### 3.3.6 Level 2: learning outcomes

Coaches generally felt they received many useful tools and beneficial training for telehealth coaching. Each of the four training units introduced some unfamiliar content, attributed to the effective delivery and well-organized work materials. Practice coaching was particularly instructive, helping coaches internalize their learning.

Coaches identified strongly with the HAPA model, especially remembering the tools they developed and presented. The importance of asking open-ended questions and allowing space for dialogue from the MI unit was well internalized. The three basic needs of the SDT were also highlighted as significant for coaching sessions. For each training unit, memory gaps were identified for most coaches.

#### 3.3.7 Level 3: application in everyday work

Coaches felt secure using the learned tools in the coaching sessions. They would frequently use the HAPA tool *Decision Balance*, goal-setting, and routine-establishing tools. Three coaches used regularly three self-efficacy tools and felt confident in their tool choice. The observation sheet served as a memory aid during sessions. The tools were used intuitively and seen as a flexible guide.

Regarding MI, coaches adopted important coaching rules previously overlooked, finding them profitable in initial sessions. A coach found the content exciting and beneficial, but noted that actual implementation depends on the coachee*:* “Sometimes I think the training was great, the content we learned was great, the tools we got were exciting, but there are always conversations where I have the impression it does not implement so well in real cases, at least not as it should” (Interview 4).

Although the training was some time ago, coaches felt well prepared for the first sessions. At time of the interviews, a few coaching sessions had taken place, which were assessed positively by the coachees.

### 3.4 Results of the follow-up assessment

The overall knowledge score of the coaches in the follow-up varied between 66 and 90%. The MI content was best remembered with a total of 88% correct, followed by the HAPA content with 80% correct.

The overall training was averagely rated as highly informative (mean = 6.6; SD = 0.5), instructive (mean = 6.3; SD = 1), practical (mean = 6.6; SD = 0.5), and satisfactory (mean = 6.6; SD = 0.5). The nutrition training session was rated as most informative, instructive, practical, and satisfactory, followed by the MI session.

In the interviews, the participants highlighted the importance of face-to-face training, which was particularly limited due to pandemic restrictions. This mode of training was regarded as the most valuable enhancement to the program. Longer training sessions were also viewed positively, with coaches expressing a desire for more content on diverse coaching techniques. For individuals with no prior coaching experience, the training provided a sufficient foundation for their future roles.

While all training units were considered meaningful, in contrast to the evaluation survey, some participants in the interviews felt that the nutrition section was comparatively shorter and less informative than the exercise section. Additionally, participants suggested that these units could have incorporated elements of role-play and reflective exercises, which were more prominent in the other units.

The interviews emphasized that while theoretical knowledge is important, empathy remains a fundamental, irreplaceable quality in effective coaching. Coaches noted that the personality and individual characteristics of a coach are crucial in building relationships and implementing coaching practices.

Participants consistently identified HAPA as the central and most impactful component of the program. Exercises associated with HAPA were particularly appreciated.

In practice, coaches predominantly used the training content intuitively. HAPA tools were frequently mentioned as being applied seamlessly, while MI techniques and SDT principles were consciously integrated into coaching sessions. Coaches reported no perceived need for additional follow-up training, as they felt that the provided materials and sessions had equipped them adequately.

## 4 Discussion

The results of the evaluation methods suggest that this training may represent a useful and important preparation for telehealth coaching, consistent with previous findings highlighting the importance of structured, theory-based coaching training (2, 3, 31). The coaches could recall a lot of the training and apply the knowledge in coaching. However, these findings are primarily descriptive and based on a small sample size and qualitative methods. Thus, conclusion regarding effectiveness should be considered preliminary.

All three evaluations highlighted the provision of important tools for the concrete implementation of the coaching sessions and the sufficient training support. The content could be primarily memorized and reproduced. The HAPA and the MI techniques were particularly important and relevant for the coaches. The follow-up assessment emphasized the intuitive application of HAPA tools and the deliberate integration of MI techniques into conversations, confirming their centrality to coaching practice. This supports prior evidence suggesting that HAPA and MI can effectively enhance patient-centered communication and behavior change (32–36). The positive evaluation may indicate a consistent use in the coaching sessions, which is in accordance with the attributed importance of the two units. It seems useful to provide a sufficient number of different tools since one coach stated it can depend on the coachee which tools to use.

The SDT unit remains contradictory. The SDT unit showed lower scores than the other units in the knowledge test, but it was rated similarly high to the other units. The qualitative interview showed different perspectives about the importance of SDT for coaching and was described as helpful but partly seen as irrelevant. Further exploration is needed. The SDT unit played a subordinate role in the coaching, which could explain the perceived lower relevance. In the follow-up interviews, SDT content was acknowledged for its theoretical value but perceived as less critical in practice, further underscoring its secondary role in telehealth coaching. These findings partially align with previous research on health coaching interventions (4, 6), which emphasize the value of motivational interviewing and structured behavioral models like HAPA. In contrast, the lesser role of SDT in this study differs from literature that highlights its contribution to long-term behavioral change (20). This discrepancy may result from the format and brevity of telehealth coaching or coaches’ background.

The units of physical activity and nutrition also tended to score lower in the evaluation survey, echoing previous concerns about the difficulty of tailoring standardized content to diverse coach backgrounds (37). This is consistent with expressions from the interviews and from the follow-up assessment. These parts were not as relevant for the coaches due to their professional background. Participants in the follow-up highlighted that the nutrition section, while meaningful, was shorter and less informative than the exercise section. They suggested integrating role-play and reflective exercises to enhance these units, aligning them more closely with the practical and interactive design of other modules. As the topics are relevant, the knowledge adaption to the coaches’ level is important. In this respect, different areas of knowledge can be profitably complemented by peer teaching. This approach is supported by prior findings that peer-based and interdisciplinary learning can enhance training effectiveness and learner satisfaction (38, 39).

Interactive training with role plays was seen as beneficial. The practice coaching among each other was rated lower in the evaluation survey than with the actor patients. In the qualitative interviews, coaches found the practice coaching among themselves particularly useful. This suggests deeper processing may have occurred between the evaluation survey and the interviews, as coaches reflected on their experiences over time. Both practice coaching forms were rated as highly beneficial. This confirms the relevance of practice coaching for coaches (1, 6, 12, 23, 40).

Due to the COVID-19 pandemic the training was reorganized to be mostly online at short notice. Coaches in turn specifically favored in-person training. During the initial session, the coaches appreciated getting to know each other in person. The follow-up assessment reaffirmed that face-to-face interactions were considered essential for building rapport and enhancing learning outcomes, outweighing the perceived benefits of online formats. The benefits of learning and practicing together in person outweighed the benefits of online training.

### 4.1 Limitations

This study has several limitations related to its design and results.

The generalizability of the findings is limited due to the small sample size (*n* = 4 at baseline, *n* = 3 at follow-up). Only four participants were included because they were the only coaches trained and available within the scope of the P-SUP project. Future research should recruit a larger and more diverse sample to validate these initial findings.

Social desirability may have influenced responses, as interviewers were known to the participants. Recall bias is another concern, given the time gaps between the training, written questionnaires, and qualitative interviews. Some coaches mentioned insufficient feedback during practice sessions. One interview had to be redone due to technical issues, potentially altering responses as the coach gained more experience. COVID-19 restrictions delayed coaching sessions and may have affected training experiences. Individual coach personalities also impacted training and coaching experiences. Critical feedback mainly came from one coach.

### 4.2 Practical implications

Suggestions for training content and format include ensuring relevance to coaching work and understanding the project’s context. Training should match the coaches’ knowledge levels, incorporating peer teaching. Increasing the length and depth of the nutrition unit and integrating reflective exercises could enhance its relevance and impact. In-person training is preferred, though online training is more convenient. Incorporating units on empathy and interpersonal skills could address the relational aspects of coaching, as highlighted in the follow-up assessment.

## 5 Conclusion

The training was perceived as informative and insightful. The MI and HAPA units were particularly significant for coaching preparation, which was underlined by the follow-up assessment. Practice coaching with actor patients and among themselves were perceived as important and useful. Balance between theory and practice was recommended. Training was rated highly with skills and knowledge retained after 6 months. The training ensured comparable levels of knowledge and work with established methods.

Practice implications (importance of actor patients; preferring in-person training) were highlighted through interviews, which are useful for further trainings. Additionally, integrating modules on empathy and interpersonal skills could address the relational dynamics central to effective coaching.

## Data Availability

The original contributions presented in the study are included in the article/Supplementary material, further inquiries can be directed to the corresponding author.
