# Supplementary material for: Evaluation of a train-the-coach program in the context of a complex intervention for diabetes type 2 and coronary heart disease patients (P-SUP study)
Source: Front Med (Lausanne). 2025 Jul 11;12:1577341. doi: 10.3389/fmed.2025.1577341 (PMC12290412; doi:10.3389/fmed.2025.1577341)
Supplement: Supplementary file 1 [file Data_Sheet_1.pdf]

Appendix A

Level	Category	Subcategory	Sub-subcategory	Definition/Use code when	Example
**Level 1: Reaction**				Level 1: Reaction - This level evaluates how the trainees react to the training program. It measures the satisfaction of the trainees towards the content, delivery method, and instructor.	
	1.1 Challenges during the training			Coaches mentioned challenges that occurred during the training	Some of the roles played were extreme/ some were different /so / the roles played by the different coaches with us were also partly / there were some where they were very cooperative and where it was very harmonious. And then the same actor played a different role and suddenly you were in a, I'll say, carnage. It was psychological/what can I say, there were people

					who didn't want to do it at all, or who had hearing impairments. That stuck with me, it was very challenging.
		1.1.1 Different personalities		Coaches mentioned difficulties during the training due to different personalities	I could imagine that the chemistry in such exercises is not quite right in the team among the colleagues, so that it could also be difficult, yes.
		1.1.2 Dealing with challenges		Coaches mentioned how they dealt with difficulties during the training	So at some point I brought it up because I noticed that I was under so much pressure and I thought I'd just tell the group and say, but I really have to get over myself now, and that was really great. Then at some point there was a training session where they asked again who was going to start and then I gave myself a jolt and said, "Come on, you should be the first and start. And I got

					great feedback from Ms. Gawlik that she thought it was great that I dared to start and that it was also a good coaching talk and yes, of course, that is also a nice/ a nice reinforcement.
			1.1.2.1 Supervisions	Coaches mentioned supervision in the context of the training and the work that they do	We have also discussed this, because the human being does not only consist of diabetes and CHD. But of course, also like all others, who also do not have this diagnosis in large share of course also their personality, personality change show whatever could be in addition.
	1.2 Further reactions			Further reactions to the training and its effects other than satisfaction and dissatisfaction	
		1.2.1 Personal approach to the situation		Coach explains a specific personal experience	Which also had a lot to do with this actor situation and also a bit so with the own claim is to want to do right. And

					that was already that was now also not only with me topic. Yes.
		1.2.2 Anticipation		Anticipation the coach felt for the training and the job	And I was actually totally curious and very much looking forward to it, because this is a completely new area for me, too. And that was the first impulse that remained with me/ that I was very full of curiosity.
	1.3 Dissatisfaction			Reactions to the training that imply a dissatisfaction	Of course, it would always be nicer to do such trainings not online but in presence, but well, the pandemic just put a spanner in the works. I find that even nicer.
		1.3.1 Overall with the Training		Overall dissatisfaction with the training	
			1.3.1.1 Improvement proposal	Suggestions for improval regarding the dissatisfaction	
			1.3.1.2 Organisation (online-teaching)	Dissatisfaction with the organization of the training and the online format	

		1.3.2 HAPA		Dissatisfaction with the HAPA-module	
			1.3.2.1 Didactics and Method	Dissatisfaction with the Didactics and Methods regarding the HAPA-module	
			1.3.2.1.1 Too much Information at that time		I found it all very exciting but then also very much at the end.
			1.2.2.1.2 Improvement proposal		some form of deskewing might then have been helpful in the practical implementation of what was learned theoretically.
		1.3.3 Motivational interviewing		Dissatisfaction with the MI-module	
			1.3.3.1 Organisation	Dissatisfaction with the MI-module regarding organization	But this was the first digital training, so the effect of personal contact was not there.
		1.3.4. Self-efficacy and Self-detamination-theory		Dissatisfaction with the Self-efficacy and Self-detamination-theory module regarding organization	
			1.3.4.1 Didactic and Methods	Dissatisfaction with the Self-efficacy and Self-detamination-theory	That was also of course an online meeting. And also a bit of lecture

				module regarding didactic and methods	mode, I'd say. So I really have a big memory gap. I can't even say what kind of exercises we did. Maybe it was too much in quotation marks, presentation for the first time, that can happen on the screen during a presentation, that you don't feel addressed or picked up in such a way that you get involved. But that is now also just an attempt for me to explain why we are no longer so present.
			1.3.4.2 Usefullness	Dissatisfaction with the Self-efficacy and Self-detamination-theory module regarding its usefullness	That was for me / is in the memory now not such a big part in my work so important / so I would say it now times like the other stories were now.
			1.3.4.3 Organisation	Dissatisfaction with the Self-efficacy and Self-detamination-theory module regarding its organisation	That was also of course an online meeting. And also a bit of lecture mode, I'd say. So I really have a big memory gap.

					I can't even say what kind of exercises we did. Maybe it was too much in quotation marks, presentation first of all, that can happen on the screen during a presentation, that you don't feel addressed or picked up in such a way that you get involved. But that is now also just an attempt for me to explain why we are no longer so present is
		1.3.5 Diet and physical activity		Dissatisfaction with the Diet and physical activity module	
			1.3.5.1 Didactic and Methods	Dissatisfaction with the Diet and physical activity module regarding didactic and methods	As far as I remember, there has not been such a large part of personal participation, active participation. But I think that is also the nature of things through this kind of knowledge that is imparted.
			1.3.5.1.1 Suggestion for improval		I don't know if it would have been possible to

					present the exercise programmes in a simple and normal way, which the participants receive, so to speak. I have seen how this unit works because I was also present at one of the pre-tests. And then I also observed Mr. Scholl during the movement part and saw what exercises he did with the participants. Maybe when I think about it, it would have been more exciting to find out what the people do in terms of content. And then also when I talk to a coach, for example, to know what he is talking about when he says after I did this and that gymnastics last week and I had this or that problem. Then you are a bit closer to the coach. But that's not

					what this one-hour training was about.
			1.3.5.2 Organisation	Dissatisfaction with the Diet and physical activity module regarding organisation	Yes, if I'm being honest and frank, I was wondering when this training was created for us, who was being offered this training, so to speak.
			1.3.5.2.1. Suggestion for improval	Suggestion for improvement regarding the organisation Diet and physical activity	No, so I found that / I would have liked it if it had somehow also been a four-hour training. But due to the fact that it was only a very short information event, I'll call it that. It was not necessary for me
			1.3.5.3 Usefullness	Dissatisfaction with the Diet and physical activity module regarding its usefullness	But in terms of content, the training really didn't get me anywhere. It was nicely done. But I personally wouldn't have needed it from my background.
		1.3.6 Practice with the actor patients		Dissatisfaction with the practice module with the actor patients	

			1.3.6.1 Didactic and Method	Dissatisfaction with the practice module with the actor patients regarding didactic and methods	Maybe this training should have lasted longer or these sequences should have lasted longer. So maybe a little more time should have been made available.
			1.3.6.2 Dissatisfaction with colleges	Dissatisfaction with the practice module with the actor patients regarding colleges	As far as that is concerned. And like I said, it's this time, pressure/this feeling that comes from it, that the feedback is actually not so accepted, not so important. It's not put in the centre so much. It's always something that has to be done quickly, so it feels like you're not taken seriously. To change that, it's up to one person here in our team. And that is the way we communicate. Although it's about communication, we are also amazed at how little willingness there is

					to change / and that every week. For us, we've partly just come to terms with it. And therefore that would be the only thing where I would say/ but it's the same every time, also in other areas. So I'm quite open about it.
			1.3.6.3 Organisation	Dissatisfaction with the practice module with the actor patients regarding didactic and methods	
			1.3.6.3.1 pressure of time	The coaches felt that there was not enough time or they felt something was rushed	sometimes, unfortunately, there was little time for feedback and it came very quickly.
			1.3.6.3.1.1 Suggestion for improval	Suggestion for improval regarding the pressure of time	This could have been avoided by having one more appointment and then, so to speak, creating the space to have time to answer questions that come up or simply / yes more time. There was also a lot of time pressure

					that was added to the schedule and that could have been reduced,
			1.3.6.3.2 with the role	The role of the acting practice was criticized or not well liked to some extend	I know that there was a situation in a conversation with me in a role that was a bit unfortunate. I can't remember the details now, but the actor, the acting patient, he obviously didn't have one piece of information that every participant in this project and in the telephone coaching has. And that somehow led to me explaining it to him in conversation, which then led to / which then took a little longer in terms of time and the feedback I got afterwards was that I shouldn't have gone into it in such detail and I was simply irritated because it was unfortunate for the

					role. Because the actor obviously lacked information, a coach knows that beforehand, and can put that aside. So that just stuck with me a little bit. Because somehow the first conversations were like that and it was a bit unpleasant for me that it went so unhappily, but otherwise I found the roles very appealing.
		1.3.7 Practice among the coaches		Dissatisfaction with the practice module with the coaches among each other	
			1.3.7.1 Efficancy	Dissatisfaction with the practice module with the coaches among each other regarding efficancy	One can also discuss "this till to death". That's a very harsh term for it, but maybe that expresses a little bit of my feeling that I had/have there, that feeling is just enough for me right now.
			1.3.7.1.1 posed situation	The practice coaching situation and its degree	So I really think that these practice units

				of naturalness or realness	with the coaches, what we did afterwards among ourselves, that was actually something that was unplanned in the training beforehand or that was built in unplanned that we then took up on our own initiative, I would find that basically very helpful to integrate into the training.
			1.3.7.2 Improvement proposal	Improvement proposal for the dissatisfaction with the practice module with the coaches among each other	So I really think that these practice units with the coaches, what we did afterwards among ourselves, that was actually something that was unplanned in the training beforehand or that was built in unplanned that we then took up on our own initiative, I would find that basically very helpful to integrate into the training.
	1.4 Satisfaction				

		1.4.1 self-efficancy and self-determination-theory		Satisfaction with the self-efficancy and self-determination-theory	Even if I now have parts/ it's been too long now. No, I didn't really miss anything. So that was very positive
			1.4.1.1 Didactics and methods	Satisfaction with the self-efficancy and self-determination-theory regarding didactics and methods	And there, too, the method was particularly memorable and it was possible to illustrate very well what self-efficacy means, what is behind this concept in contrast to, for example, compliance or other theories.
			1.4.1.2 trainer	Satisfaction with the self-efficancy and self-determination-theory regarding the trainer	Also with Angeli, I have to say again, she built it up similarly well. It was incredibly motivating how she does it. I like the way she sets up the training.
			1.4.1.3 content	Satisfaction with the self-efficancy and self-determination-theory regarding content	I: And what about the topics of self-efficacy and self-determination theory?<br>P: That is quite interesting.
			1.4.1.4 organisation	Satisfaction with the self-efficancy and self-	

				determination-theory regarding organiaation	
			1.4.1.4.1 online/in-person traning	Satisfaction with the self-efficancy and self-determination-theory regarding online/in-person traning	I found it particularly exciting how it was implemented digitally and how it simply stimulated one to deal with this topic oneself through this digital implementation.
		1.4.2 practice among the coaches		satisfaction with the practice among the coaches	on the whole, i am satisfied with the way it went
			1.4.2.1 usefulness	satisfaction with the practice among the coaches regarding usefulness	So I really think that these practice units with the coaches, what we did afterwards among ourselves, that was actually something that was unplanned in the training beforehand or that was built in unplanned that we then took up on our own initiative, I would find that basically very helpful to integrate into the training.
			1.4.2.2 Distinction between the	Distinction between the coaching practice and	In these exercises with the actors, we

			coaching practice with the actors	the actor patient exercise	colleagues were only participants in a training course organised by Mr Vithinius or Ms Gawlick or Ms Nagiak. And when we meet among ourselves, we are only in a close circle of colleagues, so then we don't have any, I'll say it like this, no leading person, no leading authority any more, so that softens the structure a bit.
			1.4.2.3 Constructive feedback	Satisfaction with the constructive feedback during the practice coachings with each other	And then, on the meta-level, I could say something else or give feedback or, as a colleague, say again, hey, why don't you go into the right direction, why don't you use the technique? So we really gave each other honest feedback, constructive feedback. I found that very good.

		1.4.3 coaching practice with the actor patients		Satisfaction with the practice coaching with the actor patients	I: And how did you find the organisation of the exercise days?<br>P: Basically good.
			1.4.3.1 organisation	Satisfaction with the organisation of the practice coaching with the actor patients	It wouldn't be a bad thing if a training course like this were to take place again, but it wouldn't have suffered in terms of content.
			1.4.3.2 usefulness	Satisfaction with the usefulness of the practice coaching with the actor patients	They were also very helpful because you could go into a kind of coaching situation and also get feedback on it: Usually when I coach, I don't get much feedback from the person I'm coaching on whether it's good or whether it has a positive or negative effect, or what's good or what's bad about it. That's why I found this exercise very helpful. They did several of them, yes,

			1.4.3.3 different roles	Satisfaction with the different roles of the practice coaching with the actor patients	Otherwise, I actually found the roles very appealing
			1.4.3.4 gender of the actor	Satisfaction with the gender choice of the actor patients	Perhaps I should also point out that I always find it good when there is an acting patient and a female patient, i.e. both men and women, but I think that's also the way it is now, in the beginning it was always just one gender.
			1.4.3.5 didactic and methods	Satisfaction with the didactic and methods of the practice coaching with the actor patients	For me, it wasn't about new content, in fact we were all familiar with that, but I found it helpful to know exactly what the patients and participants in our project actually get to know. What are the main topics?
		1.4.4 Diet and physical activity		Satisfaction with the Diet and physical activity unit	I also found nutrition very interesting.
			1.4.4.1 Organisation	Satisfaction with the Diet and physical	So for the amount of time it took, it was perfectly fine for me

				activity unit regrading organisation	that there were no practical parts or practical exercises included.
			1.4.4.2 usefulness	Satisfaction with the Diet and physical activity unit regrading usefulness	But I have the feeling that what was taught there is a very good basis for being able to work with it in coaching. And from that point of view, I didn't miss anything in terms of previous education.
			1.4.4.3 didactics and method	Satisfaction with the Diet and physical activity unit regrading didactics and method	In principle, I found that this concept of Mediterranean nutrition with the plate concept is very simple and clearly structured and can be put into practice very well, so that I think even people who do not have this professional background can convey it well and can also integrate it well into further practice.
			1.4.4.3.1 knowledge reproduction	Coach reproduces what they had learned	What I found interesting was simply

				regarding diet and physical activity	to look at the new WHO guidelines again, because they have just been updated this year or last year.
		1.4.5 MI			
			1.4.5.1 didactic and method	Satisfaction with the MI unit regrading didactics and method	But apart from that, I can only say that I found it very well structured and the concept was conveyed well.
			1.4.5.2 reaction	Reaction to the MI unit	Overall the training was good too.
			1.4.5.3 content	Satisfaction with the MI unit regrading content	And also related to the content, very exciting.
			1.4.5.4 usefulness	Satisfaction with the MI unit regrading usefulness	I also found it very exciting in principle. Also very useful
			1.4.5.5 organisation	Satisfaction with the MI unit regrading organisation	
			1.4.5.5.1 online-training	Satisfaction with the MI unit regrading online training	And then we worked with our colleagues in a breakout session and got together again. I thought that was good. I thought it worked surprisingly well. If you've never done this

					before and you're new, you're often first of all / how does it work when you're used to being present, of course it's much nicer. But it worked amazingly well and I didn't think there was any difference in the content that came across. So that was completely okay.
		1.4.6 HAPA		Satisfaction with HAPA	Yes, specifically I don't know what else to do now / specific parts / as a whole I found it a very good training.
			1.4.6.1 organisation	Satisfaction with HAPA regarding organisation	Yes, I have basic/ I can't really distinguish between the individual ones now, but this part was the basis and the foundation of it and I knew beforehand /in itself and what it's about and also actually what the study is fundamentally about and what the project is about.

			1.4.6.2 didactic and method	Satisfaction with HAPA regarding didactic and method	HAPA model. Well, the HAPA model is very complex, but it is also important to recognise how I can actually identify exactly where a coach stands, i.e. where I can pick them up. And what steps can I then take with them? Exactly, so I think that's enough about the HAPA model for now.
			1.4.6.3 usefulness	Satisfaction with HAPA regarding usefulness	Of course, we were also given case examples in small groups, or we were presented with exact patient cases, which we were then supposed to assign to the individual groups. So into unprepared, prepared and active. I found that very helpful and useful.
			1.4.6.4 praise of the HAPA tools	Satisfaction with HAPA tools	So I find the tools basically a helpful guide.
			1.4.6.5 online traning	Satisfaction with HAPA regarding online traning	It was basically very good. That was the only

					training in presence form.
		1.4.7 overall the training		Satisfaction with the overall traning	In general, really very very good
			1.4.7.1 didactics and methods	Satisfaction with overall traning regarding didactic and method	But apart from that, I can only say that I found it very well structured and the concept was conveyed well.
			1.4.7.1.1 Team	Satisfaction with the team	Because we also got to know each other much better through this practice,
			1.4.7.1.1.1 personality of the coach	Positive comments regarding the personality of the other coaches	Because through this practice we also got to know each other much better, both personally but also to know where one or the other has their strengths, so for example I was able to take a lot from the calmness of (name). So we realised that we are all really four very different types.
			1.4.7.1.2 trainer	Positive comments regarding the trainer	Later, we had other training sessions, also with Angeli, and they

					were all very good. Very interactive!
			1.4.7.1.2.1 support offer	Positive comments regarding the support offer	Oh, yes, sufficient again and again. We all know that / or I know that I can call Ms Nagiak or Ms Gawlik at any time and write to them and we can organise a Zoom meeting if necessary. We have used this for smaller questions several times and always get a relatively quick response, which is also good to know. So I don't have the feeling that I'm somehow sitting alone in the home office and I have problems and don't know who I can contact because I don't know if there is individual feedback.
			1.4.7.1.3 practical application	Positive comments regarding practical application tasks in the training	We then learnt in theory to simply try it out with practical exercises. I think that

					was a very important part of it.
			1.4.7.2 no improvements needed	Overall satisfaction and no improvement needed	Both in presence and in online form with actor-patients. Even though I am not supposed to mince my words, I really can't say anything negative about it, not even superordinately.
			1.4.7.3 usefulness	Overall usefulness	I enjoyed the training very much and was able to learn a lot from it.
			1.4.7.4 content	Overall satisfaction with the content of the training	So overall, I actually experienced it as positive. I found the content very different.
			1.4.7.5 organisation	Overall satisfaction with the organisation of the training	But the question is still how uniform something like that becomes, and especially within the framework of a research project, I think it must somehow have a structure, a guideline. And that's why, as I said, I rate the training overall as positive, as pleasant. And I am also pleased that we were

					informed so comprehensively.
			1.4.7.5.1 online/in-person training	Overall satisfaction with the online/in-person training aspect of the training	In and of itself, the content was also conveyed really well via the video conferences.
		1.4.8 Inclusion of the coaches in the overall course of the project		Mentioning of the inclusion of the coaches in the other component of the overall project	For me, it wasn't about new content, in fact we were already all familiar with it, but I found it helpful to know exactly the same thing: what do the patients and participants from our project actually get to know?
**2. Learn success**					
	2.1 new content				
		2.1.1Diet and physical activity		New content regarding Diet and physical activity	With the self-determination theory, that was new to me.
		2.1.2 HAPA		New content regarding HAPA	It was very exciting because the HAPA model was new to me.
		2.1.3 Acting practice		New content regarding acting practice	I have never done exercises like this with actors before
		2.1.4 Motivational Interviewing		New content regarding MI	But that was actually a completely new area that I didn't know at all

					and that was also a personal enrichment for me.
	2.2 Memory gaps			Coach shows memory gaps tough saying e.g. that he/she doesn’t remember something	
		2.2.1 Motivational Interviewing		Memory gaps regarding MI	To be honest, I've just found the training courses a bit blurred, because some of them took place online.
		2.2.2 Diet and physical activity		Memory gaps regarding Diet and physical activity	Yes, I am really thinking about what we have done there.
		2.2.3 Self-efficacy and self-determination-theory		Memory gaps regarding Self-efficacy and self-determination-theory	I:.....can you remember how you felt at that time compared to the training, maybe some aspects of the content without remembering the whole training?<br>P: No, unfortunately not. It is a bit uncomfortable for me right now, but unfortunately no.
		2.2.4 HAPA		Memory gaps regarding HAPA	I really realise that the training was a bit ago.

	2.3 Learn success self efficacy			Progress in knowledge or expertise regarding the self efficacy unit	that has become very solidified as a theory in my head.
	2.4 Learn success coaching practice exercises			Progress in knowledge or expertise regarding coaching practice exercises	At the beginning, of course, you had the impression that you really had to implement a tool in terms of content, but over the course of the next few weeks and training sessions and the practical exercises, it became clear that everyone has their own individual leeway and it is only a guideline. No, I can identify with it / could identify with it very quickly.
		2.4.1 Practice with the actor patients		Progress in knowledge or expertise regarding coaching practice exercises with the actor patients	And sometimes it is perhaps also good to have this extreme stress. Because it might have been produced because it wasn't optimal, but nevertheless it might have worked within this greater stress and you

					could see and see how I deal with it. And that's why I tended to take the positive out of it and say that's just the way it is. Then I also had to show or see for myself what I could do. Then that is also completely okay for me.
		2.4.2 Practice with the coaches		Progress in knowledge or expertise regarding coaching practice exercises among the coaches	oh yes, you should only do it that way. These are the theories, this is what we want to implement. Then we try to do that in the discussions when we practise with each other. Then all of a sudden it's just there.
	2.5 Learn success motivational interviewing			Progress in knowledge or expertise regarding the MI unit	For example/ in the beginning, now it has really improved, I found it extremely difficult to ask open questions.
		2.5.1 Off-the-job learning success		Progress in knowledge or expertise regarding the personal life	No, except really thank you again! And that it was a lot of fun for me professionally, but also

					in terms of transferring it to private communication, and that I can get an incredible amount out of it.
	2.6 Learn success HAPA			Progress in knowledge or expertise regarding HAPA	As I said, the model was new. But I identified with it very quickly/ I personally can also identify very well with how I work, or how I have worked with patients so far, and I had the feeling relatively quickly that this is a tool with which I can work very well. And also with the tools in terms of content. I think they are all actually very good. At the beginning, of course, you had the impression that you really had to implement a tool in terms of content, but over the course of the next few weeks and training

					sessions and the practical exercises, it became clear that everyone has individual room for manoeuvre and that it is only a guideline. No, I can identify with it / could identify with it very quickly.
		2.6.1 HAPA tools		Progress in knowledge or expertise regarding HAPA	For me, the most important thing is not that I can implement all the tools in theory, so to speak, in my sleep, but rather that I feel really confident for the first third or the first half of the tools, thirds are too few, that half of the tools, that is, I feel really confident and can build on them.
			2.6.1.1 Implementation difficulties		I: how confident do they feel in using the tools?<br>P: It varies, so I have to say that this has a bit to do with the role I was given in our exercises. I

					didn't really have the opportunity to go through all the tools in a practical exercise. But it was important for me to go through the first tools again and again so that they really sit. Assuming that we don't only have active people assigned to us, that's different in my case for the time being, but that's not a bad thing at all. In this respect, I have not really been able to practise all the tools in such detail in practical exercises.
	2.7 Interview was helpful			Progress in knowledge or expertise thorugh the interview	For me, recapitulating that now and going back in there and so on is also a learning process. Because you call up a lot of things. So I thought it was a very good idea and it was great that we did it. Helpful!

	2.8 Knowledge rendition			Coach says something that shows that they retained the information of the training	(thinking) What I still remember very well is that it was structured in such a way that these three aspects of paying attention to the needs of the coaches and also preserving the autonomy of the coach and the need for social connection have strengthened this theory in my mind. And that this is also a very important aspect/three very important aspects that one should pay attention to in coaching in order to be able to work successfully.
**Level 3: Behavior**				Level 3: Behavior - This level evaluates the changes in the trainees' behavior as a result of the training. It assesses whether the trainees apply their newly acquired knowledge and skills on the job.	

	3.1 Application in the workday			Coach mentions and or explains that they applied knowledge and know-how from the training in the coaching sessions	So, in addition to the pools, I also prepared some posters myself, which I can then stick on my laptop or something, which is why I felt very well prepared and have also had really funny conversations so far.
		3.1.1 challenges		Challenges that occur while applying the learned in the workday	Sometimes I think the training was great, the content we learned was great, the tools we got were very exciting, but there are always conversations where I have the impression that it is not as good in real life as it should be in my imagination, which would meet my requirements.
			3.1.1.1 Dealing with challenges	The dealing with the challenges of the application of the learned in the workday	Oh, that was quite good. Because I have a very talkative first coach. I had to make an agreement with him right at the first meeting that we had to

					watch the time and that I could interrupt him or that he would feel hurt. He gave me a lot of space to really consciously make sure that I /that I think about my questions carefully, that I get as much information as possible with as short a question as possible. And I was also really really satisfied with these open questions and /that because I had the impression that I really succeeded quite well in the first two interviews I had. But also because of the information and the training that I received in advance.
			3.1.1.2 With the HAPA tools	The dealing with the challenges of the application of the learned HAPA Tools in the workday	Yes, one tool that I often use is the decision balance. I find it more difficult with the health motivation tool, or with the self-efficacy tools, which I also find more

					challenging, I notice that. It is often more difficult for me to communicate this. One tool I often use is my routine or my goal.
		3.2.1 Subjective competence conception		The coach shows their perceived level of competence	And in this respect/ so I felt very well prepared I was a bit excited from the first interview then it really started.
			3.2.1.1 With motivational Interviewing	The coach shows their perceived level of competence with the MI	How does the implementation of the aspects of motivational interviewing work? P Well, I think it's very easy for me.
			3.2.1.2 With the HAPA tools	The coach shows their perceived level of competence with HAPA	I had the feeling relatively quickly that this is a tool I can work with very well. And also with the tools in terms of content. I also find them all very good.
Other					
	4.1 Pre-experience			Mentioning the pre-experience the coach has	
		4.1.1 PSG-Project		Mentioning the pre-experience regarding	Also on the nutrition part, I saw excerpts of

				the PSG-Projekt the coach has	this part of the training in the pre-tests, if that hadn't been the case, I could imagine that telephone coaching sessions that don't focus on nutrition would also be a small impulse for us.
		4.1.2 Diet and physical activity		Mentioning the pre-experience regarding the Diet and physical activity the coach has	it was / or is of course not my main topic as a sports scientist, so it was fundamentally different from this movement part. For me, it wasn't about new content - in fact, we were all familiar with it.
		4.1.3 Motivational interviewing		Mentioning the pre-experience regarding the motivational interviewing the coach has	But for me, in essence, not much new. There were maybe two more aspects where I thought, ah, yes, okay, I haven't looked at that side yet either. All in all, it was perfectly okay due to the training, but not so much new for me.

		4.1.4 Self-efficacy and self-determination-theory		Mentioning the pre-experience regarding the self-efficacy and self-determination-theory the coach has	But that might also be because it wasn't necessarily something completely new, but because I had added a bit of content to it and what we had already done before was something that I had somehow already practised a bit until then, as I just told you, in private but also with my colleagues.
		4.1.5 HAPA		Mentioning the pre-experience regarding HAPA the coach has	Yes, actually yes. I think most of us actually knew the HAPA model. So it was simply a refresher for many of us. But overall I had the feeling that all of us who came had a basic idea of what the HAPA model is and what it can do. Yes, and then simply intensified it again.
		4.1.6 unspecific		Mentioning an unspecific pre-experience coach has	I think the basis that I brought with me could be so great somehow

					especially in this area of coaching again /so much could be built on /
	4.2 Supervision in the everyday work			Supervision	Of course, we talked again about how to deal with / have supervisions. And during the coaching, when they really start, we have the possibility / and these are supposed to be fixed supervision appointments where you can report with such coaches about how you can deal with it.
